# Ovarian Reserve and Hormone Alteration Following Laparoscopic Ovarian Drilling for Polycystic Ovarian Syndrome: A Systematic Review

**DOI:** 10.7759/cureus.62092

**Published:** 2024-06-10

**Authors:** Ethan Slouha, Jeremy Segal, Colton White, Theresa Pallotta, Shanalyn Ghosh, Lucy A Clunes, Theofanis F Kollias

**Affiliations:** 1 Pharmacology, St. George's University School of Medicine, True Blue, GRD; 2 Pharmacology, St. George’s University School of Medicine, True Blue, GRD; 3 Microbiology, Immunology, and Pharmacology, St. George's University School of Medicine, True Blue, GRD

**Keywords:** ovarian hormones, ovarian reserve, laparoscopic ovarian drilling, laparoscopic surgery, polycystic ovarian syndrome

## Abstract

We aimed to conduct a systematic review of the data in the literature on ovarian reserve and ovarian hormone following laparoscopic ovarian drilling (LOD). The PubMed, ScienceDirect, and ProQuest databases were comprehensively searched using a combination of keywords such as “ovarian reserve”, “laparoscopic ovarian drilling”, “luteinizing hormone”, “follicle-stimulating hormone”, “inhibin”, “LH/FSH ratio”, “ovulation”, and “testosterone”. All studies involving females of reproductive age who were officially diagnosed with polycystic ovarian syndrome (PCOS) and had undergone LOD with reported data concerning at least one of the following parameters were considered for inclusion: ovarian reserve, anti-Mullerian hormone (AMH), inhibin, follicle-stimulating hormone (FSH), luteinizing hormone (LH), LH/FSH ratio, and testosterone.

All the included studies were evaluated by the GRADE scale for bias and their findings were synthesized by four independent coauthors. A total of 38 studies involving 3118 female patients were included. Based on our findings, a significant number of participants experienced spontaneous ovulation along with a significant decrease in ovarian reserve, and a significant decrease in AMH, LH, and testosterone, with no significant changes in FSH and inhibin B. With the end goal of LOD being to improve fertility and pregnancy rates among females with PCOS, it is important to look at the first few steps that enable this. As expected, there was a significant improvement in ovulation while the ovarian reserve decreased. Along with the decrease in ovarian reserve, there was a significant normalization in AMH, LH, and testosterone levels. LOD may exert its main effects through the manipulation of the ovarian reserves.

## Introduction and background

Polycystic ovarian syndrome (PCOS) affects up to 15% of females and is considered one of the most common endocrine pathologies in females of reproductive age [1]. While PCOS is generally a diagnosis of exclusion, the definitive diagnosis is made based on the Rotterdam criteria, consisting of at least two of the following: hyperandrogenism (clinical or biological), polycystic ovary morphology, and anovulation [2]. PCOS is defined as a hyperandrogenic state, typically stemming from the ovaries, due to an exaggerated response of 17-hydroxyprogesterone to gonadotropin stimulation [1]. This dysregulation has been posited to be due to excess insulin, which sensitizes the ovary to luteinizing hormone (LH), thereby leading to the overproduction of downstream steroid hormones such as testosterone [2].

The androgens ultimately lead to the formation of polycystic ovaries as they recruit primordial follicles and initiate premature luteinizations, impairing the dominant follicle selection [1]. The dysregulation also alters the pulsatile release of gonadotropin-releasing hormone, decreasing follicle-stimulating hormone (FSH) simultaneously and increasing the LH/FSH ratio [2]. The first-line intervention for PCOS involves lifestyle modifications with the goal of weight loss and glucose control [1]. When this fails, pharmacological treatments such as hormonal contraceptives, metformin, and infertility treatment with clomiphene citrate are used [1]. In some cases, however, more aggressive treatment needs to be employed, such as laparoscopic ovarian drilling (LOD).

LOD is primarily based on the laparotomic “wedge resection” procedure of the ovaries to aid in treating ovulation to improve the chances of pregnancy [3]. While this surgery is no longer routinely performed due to drastic improvements in pharmacological management, medications may prove to be refractory in some cases. The mechanism of LOD is through the mechanical manipulation of the ovary and its follicles, either by heat or laser, but the reason for subsequent follicular growth followed by ovulation has not been elucidated [3]. The proposed pathophysiology centers around this mechanical manipulation of the ovarian follicle via thermal destruction, leading to a significant decrease in most local ovarian hormones and androgens [3]. At the same time, this normalizes the intrafollicular environment to allow for follicular maturation and ovulation due to the rise in FSH [3,4]. The thermal injury also causes an increase in ovarian blood supply, which allows for the cascade of local growth factors to grow the follicle and, therefore, ovulation [3]. Also, another possible mechanism behind LOD's success is the supposed reduction in anti-Mullerian hormone (AMH) concentrations and disruption of the polycystic ovary walls [3].

Some studies have found no significant difference between LOD and clomiphene citrate in terms of restoring ovulation [3]. LOD has been shown to improve fertility in up to 64% of females who have refractory PCOS despite being on clomiphene citrate, mainly because of the regulation of abnormal menstrual cycles due to hormonal alteration [3]. One or two punctures are insufficient to evoke ovulation; studies have reported that four or more are necessary to lead to success regarding ovulation and even fertility rate; essentially, the success rate is positively correlated with the energy used [3]. Among specific concerns associated with LOD is the iatrogenic creation of adnexal adhesions, which can lead to complications in pregnancy, such as ectopic pregnancy and reduction in the ovarian reserve, affecting future fertility [3]. New techniques have emerged to improve the LOD, such as unilateral ovarian drilling (ULOD), as it still induces activity in both ovaries while minimizing procedure time [3]. One thing is absolutely evident: LOD is not a permanent solution, as its effects only last for several years, which may be why it is perfect for periods when fertility is sought [3,5].

This review focuses on menstrual patterns following LOD, its effects on the ovary and local ovarian hormones, and how they contribute to changes in the menstrual pattern. We aim to evaluate the alterations in the menstrual cycle following LOD and highlight the changes in the ovarian reserve, AMH, inhibin B, LH, FSH, LH/FSH ratio, and testosterone.

## Review

Methods

Study Design

This systematic review was conducted with strict adherence to the Preferred Reporting Items for Systematic Reviews and Meta-Analyses (PRISMA) guidelines [6]. Two independent coauthors performed the literature search through PubMed, ScienceDirect, and ProQuest, which initially yielded 14,457 publications. Keywords used consisted of a combination of “ovarian reserve”, “laparoscopic ovarian drilling”, “luteinizing hormone”, “follicle-stimulating hormone”, “inhibin”, “LH/FSH ratio”, “ovulation”, and “testosterone” along with boolean operators "AND" and "OR", with the last search being conducted in April 2023.

Criteria for Study Selection

The search was further narrowed down by coauthors based on the following inclusion criteria: studies performed on humans; published between 2003 and 2023; full-text availability; explicit focus on the outcomes of LOD with respect to ovarian reserve, ovulation, or hormones; and peer-reviewed interventional or observational studies (i.e., clinical trials, cohort studies, case series studies, cross-sectional studies). Articles were excluded if they were duplicates, not in English, and involved a combination treatment method as their intervention. This review has been registered with the International Prospective Register of Systematic Reviews (CRD42024504304).

Assessment of Risk of Bias

Due to the significantly small sample sizes of the studies used, as well as certain regional variations observed, a high risk of bias was initially appraised by one coauthor. The other coauthor concluded that there was a moderate risk of bias due to explicit methodologies in a significant amount of the studies and replication of methods between studies. A final coauthor evaluated the articles and determined that the risk of bias was moderate due to the influence of methodology outweighing somewhat smaller sample sizes with respect to effect size in some studies. All three authors evaluated bias through the GRADE scale. However, results should still be interpreted carefully.

Data Extraction and Synthesis

Four independent coauthors extracted information from each article and input the information into a formatted Excel sheet to track specific values. In the same sheet, they also included the overall thought or idea each article was trying to convey concerning each result. A final author reviewed the extracted information while extracting their interpretation of the result. This author then compiled an outline synthesizing the agreed-upon result and the final, consensus-based output when conflict was present between the other coauthors.

Results

Study Selection

**Figure 1:**
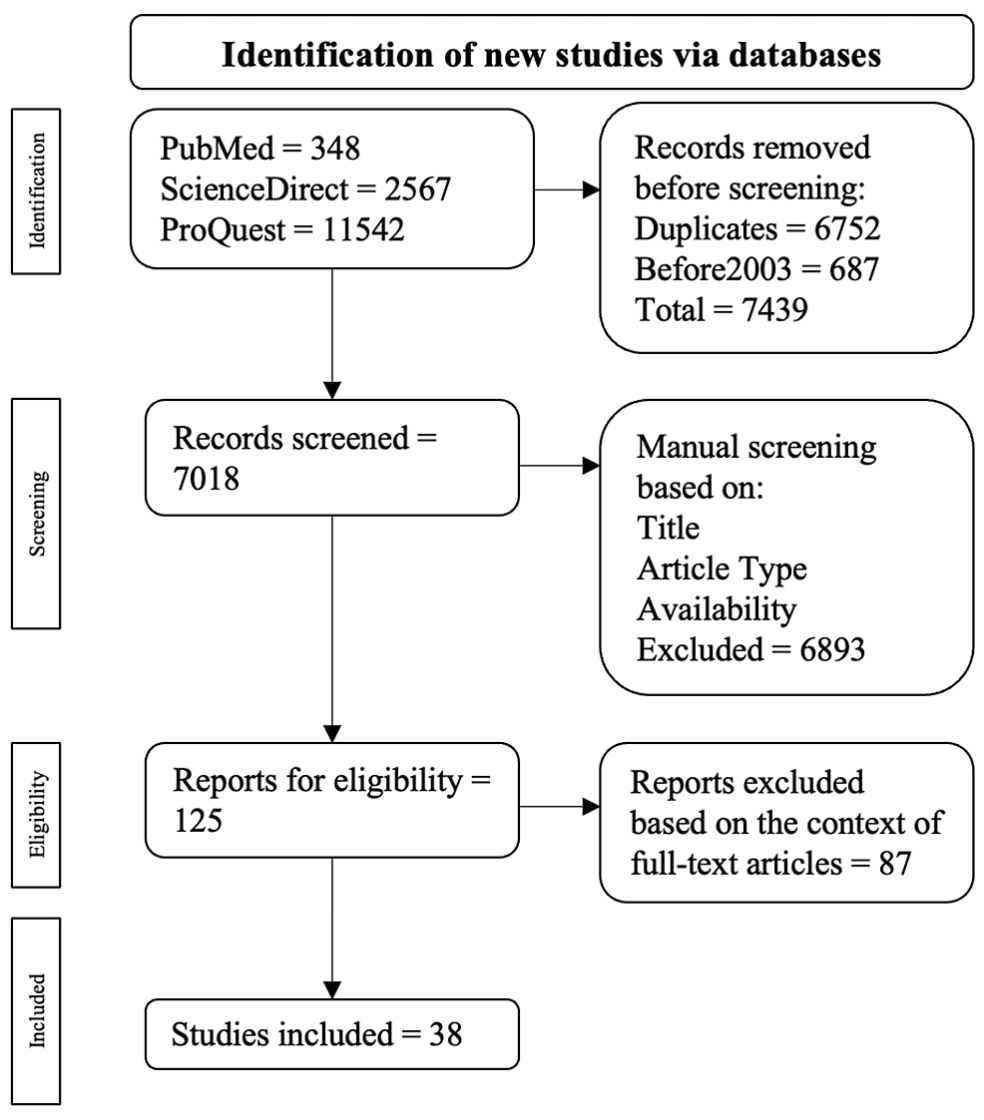
Prisma flow diagram illustrating the study selection* ^*^[6] PRISMA: Preferred Reporting Items for Systematic Reviews and Meta-Analyses

Two independent authors identified 14,457 publications from 2003 to 2023 through PubMed, ScienceDirect, and ProQuest databases. The screening process followed the PRISMA workflow; 6752 duplicates were removed and 687 articles were excluded as these were published before 2003; 7018 publications underwent manual screening based on their title, study type, study participants, and abstract to ensure those selected fit our criteria, and were narrowed down to 125 articles. Four independent coauthors evaluated the remaining articles based on full-text analysis. Ultimately, a total of 38 studies were selected for analysis. Figure 1 shows the PRISMA flow diagram depicting the study selection.

Study Characteristics

Twenty cohort studies, three cross-sectional studies, two case series, and 12 randomized control trials were included in this systematic review. No study covered all components or hormones that formed part of our intended analysis but involved at least three to five primary outcomes. No study reported a long-term follow-up of PCOS over six months, but about half of the articles included pregnancy rate, which was not used in this review we focused on the direct effects of LOD. In all patients, PCOS was diagnosed based on Rotterdam criteria and supported by pre-LOD laboratory reports (Table 1).

**Table 1:** Characteristics of the included studies LOD: laparoscopic ovarian drilling; PCOS: polycystic ovarian syndrome; AMH: anti-Mullerian hormone; AFC: antral follicle count; FSH: follicle-stimulating hormone; LH: luteinizing hormone; OHSS: ovarian hyperstimulation syndrome; OV: ovarian volume; BLOD: bilateral laparoscopic ovarian drilling; BMI: body mass index; ULOD: unilateral laparoscopic ovarian drilling

	Author	Country	Design and study population	Findings	Conclusions
1	Abulkassem et al., 2021 [7]	Egypt	Prospective nested cohort Study (n = 38)	A significant negative correlation was present between age and pre- and post-LOD AMH levels. Amenorrhea was associated with a greater difference in AFC between pre and post-LOD of the right ovary. LOD led to a significant decrease in serum AMH, AFC, and ovarian volume, indicating a reduction in ovarian reserve. There was a notable increase in FSH levels post-treatment	In females with PCOS, LOD significantly decreases serum AMH, AFC, and ovarian volume while increasing serum FSH. Amenorrhea was associated with a greater difference in AFC between pre and post-LOD of the right ovary
2	Al-Ojaimi, 2004 [8]	Bahrain	Prospective cohort study (n = 181)	After LOD, spontaneous ovulation typically occurred in obese subjects with elevated initial LH and LH/FSH ratios. All experienced notable reductions in LH, LH/FSH ratio, testosterone, and increased FSH levels. The changes in LH and LH/FSH were more pronounced in responders, particularly those who were obese	LOD is a successful intervention for females with polycystic ovarian syndrome who do not respond to clomiphene citrate, resulting in significant hormonal shifts
3	Amer et al., 2007 [9]	UK	Prospective interventional study (n = 50)	Females who were not obese had significantly higher levels of inhibin B concentration than obese women. 78% of patients ovulated following LOD with no significant change in inhibin B concentration	LOD allowed for improvement in ovulations without changes to inhibin B
4	Api et al., 2005 [10]	Turkey	Prospective, non-randomized, controlled trial (n = 45)	Post-LOD, 93.3% had regular cycles, significantly reducing T, free T, LH, and LH/FSH ratio	LOD could potentially lower the risk of OHSS and multiple pregnancies compared to gonadotropins while maintaining a similar rate of conception success
5	Azzam et al., 2019 [11]	Egypt	Prospective comparative clinical trial study (n = 48)	There were decreased levels of AMH, LH/FSH ratio, and inhibin B after LOD at 3 and 6 months	AMH levels may be useful in predicting the results of LOD in those with PCOS and resulting pregnancy post-operation
6	Beltadze and Barbakadze 2015 [12]	Georgia	Cross-sectional study (n = 56)	Females with PCOS who underwent LOD treatment exhibited lower levels of AMH and higher levels of FSH compared to those who underwent conservative treatment. Additionally, these females demonstrated fewer antral follicles and a smaller mean ovarian volume than their counterparts	Females with PCOS who underwent non-invasive treatment have better ovarian reserve compared to the females who underwent invasive treatment in adolescence
7	Debras et al., 2019 [13]	France	Retrospective cohort study (n = 289)	Post-LOD success is linked to normal BMI, <3 years infertility, AFC <50, age <35. Of 33 undergoing second drilling, 57.6% conceived, 52.6% naturally. Effective with prior success or high AFC (over 55) failure	LOD enabled females with PCOS to achieve spontaneous pregnancies. This surgery potentially allows for multiple pregnancies over time
8	Elmashad, 2011 [14]	Kuwait	Prospective controlled study (n = 43)	Following LOD, both AMH levels and blood flow measurements in the PCOS group decreased markedly. Females in the PCOS group who successfully ovulated post-LOD had lower pre-surgery AMH levels than those who did not respond to the treatment. Additionally, a strong positive relationship was observed between AMH levels both before and after the LOD procedure within the PCOS group	Measuring AMH levels in females with anovulatory PCOS is a good indicator in predicting the effectiveness of LOD
9	Elnaggar et al., 2016 [15]	Egypt	Prospective interventional study (n = 50)	Following LOD, prolactin, LH, and testosterone concentrations significantly decreased, while FSH levels remained unchanged after the procedure	LOD improves pregnancy rate and may be an optimal second-line treatment for PCOS
10	Farzadi et al., 2012 [16]	Iran	Cross-sectional study (n = 30)	AMH decreased by 8.3% 6 months following surgery, with a 6.6% decrease in LH and 11.1% in mean serum testosterone	LOD may be used to improve the reproductive rate in females with PCOS
11	Gafaar et al., 2018 [17]	Egypt	Case series study (n = 30)	63.3% of patients developed spontaneous ovulation following LOD, while AMH levels decreased significantly by 16%. It is possible that pre-LOD AMH levels can predict the outcome of LOD	LOD was shown to lead to the resumption of ovulation while decreasing AMH levels
12	Hafizi et al., 2020 [18]	Iran	Randomized controlled trial (n = 60)	One month following LOD, there was no significant difference in AMH or testosterone levels. However, there was a significant difference in AFC and testosterone between standard LOD and dose-adjusted LOD, where standard showed a greater decrease, and AMH showed a greater decrease in dose-adjusted LOD. Ovulation and cycle regularity did not differ between the groups	There is no difference between the use of standard and dose-adjusted LOD.
13	Hamed et al., 2010 [19]	Egypt	Randomized controlled trial (n = 110)	There was a significant decrease in testosterone and LH levels following LOD, with higher rates of ovulation, regular cycle, and increased pregnancy rate compared to metformin alone	LOD may be a superior treatment method for PCOS as it restores ovulation and pregnancy potential
14	Hendriks et al., 2014 [20]	Netherlands	Prospective controlled study (n = 21)	Within hours following surgery, there was a significant increase in FSH, LH, and estrogen, with a decrease in testosterone and AMH. In the days following, testosterone and AMH remained significantly lower than baseline	LOD may be used to treat refractory PCOS and improve fertility
15	Masroor et al., 2022 [21]	Iran	Prospective cohort study (n = 78)	There was a statistically significant decrease in oligomenorrhea following LOD. There was also a statistically significant decrease in AMH following LOD	AMH could be useful in predicting the success of LOD
16	Kamal et al., 2018 [22]	Egypt	Prospective cohort study (n = 80)	Following LOD, both ovaries showed a significant decrease in AMH, AFC, OV, and vascular indices	LOD proper alters ovarian reserve parameters and blood flow to semi-appropriate levels
17	Kandil et al., 2018 [23]	Egypt	Randomized clinical trial (n = 122)	This study showed a significant average overall decrease in AMH and ovulation following LOD	LOD can potentially be an effective treatment for lowering AMH and increasing ovulation in women with PCOS
18	Kandil et al., 2005 [24]	Egypt	Prospective cohort study (n = 20)	There was a significantly reduced average quantity of inhibin B following only BLOD. There was also a significantly reduced ovarian reserve with AFC and a subsequent increase in ovulation in the women studied	Inhibin B could potentially be a sign of decreased ovarian reserve and AFC
19	Kucuk and Kilic-Okman, 2005 [25]	Turkey	Prospective clinical trial (n = 22)	Statistically significant decreases in LH, testosterone levels, and LH/FSH ratio following LOD. Significant improvement was noted with the regularity of menses	For treating patients with PCOS, LOD may be effective
20	Liu et al., 2015 [26]	China	Randomized controlled trial (n = 141)	There was a statistically significant decrease in LH and LH/FSH ratio following LOD	LOD can statistically significantly decrease LH levels and LH/FSH ratio in individuals with PCOS
21	Mohamed et al., 2019 [27]	Egypt	Prospective clinical trial Study (n = 40)	A significant decrease in AMH and LH/FSH ratio was found after 3 months following LOD. Patients with increased AMH levels before the LOD procedure had an increased potential for pregnancy. This may suggest that minor AMH elevated patients may not fully improve their chances of pregnancy with LOD. Mean inhibin, AMH, and LH/FSH levels significantly decreased following LOD	AMH can be a significant predictor of post-LOD procedure ovarian reserve, potentially, as well as LOD results concerning PCOS patients. Overall rates of pregnancy had a significant relation to AMH levels following the operation
22	Ogawa et al., 2021 [28]	Japan	Retrospective cohort study (n = 60)	Following LOD, there was a 70% change in AMH levels for patients who had > 10 ng/ml preoperatively, significantly higher than patients with a preoperative AMH level of < 10 ng/ml who had a 63% change. Patients with a BMI < 18.5 kg/m^2^ had a significantly increased percent change of AMH levels compared to those with BMIs between 18.5-25 kg/m^2^	Decreases in AMH levels per puncture were higher in patients with higher levels of AMH preoperatively and lower BMIs
23	Paramu, 2016 [29]	India	Prospective cohort study (n = 30)	Both responders and non-responders were noted to have a significant decrease in their AMH levels postoperatively	AMH measurements can predict the outcomes of LOD in these patients
24	Rezaei et al., 2020 [30]	Iran	Prospective cohort study (n = 35)	There was a significant increase in FSH levels and a decrease in AMH levels for both patient groups (ULOD and BLOD). While no significance was noted between groups for the decrease in AMH levels, a statistical significance was found between groups in FSH level increases	For PCOS patients who are clomiphene-resistant, post-operative levels of AMH declined while FSH levels increased, which suggests a decrease in these patients’ ovarian reserve
25	Rezk et al., 2015 [31]	Egypt	Randomized clinical trial (n = 105)	There was a significant decrease in AMH levels in both ULOD and BLOD groups after 3 and 6 months, with no significant difference between groups. There was also a significant decrease in AFC levels in both ULOD and BLOD groups after 3 and 6 months, with a significantly higher AFC level noted in the ULOD group after 6 months	AMH and AFC levels decreased significantly after ULOD and BLOD in 3 and 6 months. ULOD patients retained higher AFC after 6 months vs BLOD patients
26	Roy et al., 2010 [32]	India	Randomized clinical trial (n = 43)	Testosterone and LH levels decreased significantly after ULOD. However, there was no significant decline in FSH levels after ULOD	For PCOS patients who are clomiphene-resistant, Rosiglitazone and CC therapy might be just as effective as ULOD
27	Salem et al., 2017 [33]	Egypt	Prospective cohort study (n = 37)	A significant decrease was noted in LH, AMH, AFC, and testosterone, specifically six months after LOD procedures	For PCOS patients who are clomiphene resistant, monitoring the levels of AMH and AFC can predict LOD outcome and ovarian reserve
28	Seyam et al., 2014 [34]	Egypt	Prospective controlled study (n = 70)	Statistically significant differences were noted in the decrease in AMH levels and AFC before and 6 months after LOD procedures in the LOD group vs the CC group	Outcomes of LOD procedures may be assessed using the AMH and AFC levels in women with anovulatory PCOS, which can help determine the ovarian reserve
29	Sinha et al., 2019 [35]	India	Cross-sectional study (n = 50)	Testosterone, LH, and LH/FSH ratio were reduced after LOD. FSH was increased after LOD. Hirsutism and acne were reduced post-LOD, and menstruation became regular in those with PCOS	LOD has been shown to reduce the testosterone and LH/FSH ratio
30	Sunj, et al., 2014 [36]	Croatia	Prospective cohort study (n = 96)	AMH, testosterone, and LH levels were significantly decreased post-diathermy. The bilateral group showed increased LH levels in the month 1 follow-up. AMH levels decreased at month 1 and month 6 follow-ups in the unilateral group. Baseline testosterone and testosterone levels at month 1 post-op were significant	Testosterone levels were found to be an appropriate indicator for ovulatory response after diathermy
31	Weerakiet et al., 2007 [37]	Thailand	Cross-sectional study (n = 21)	FSH was significantly higher in the LOD group. AFC levels were lower in the LOD group. AMH levels were lower in the LOD group versus the PCOS group, but this was not statistically significant	The ovarian reserve was lower in the LOD than in the PCOS group. PCOS with and without LOD had greater ovarian reserve compared to those with normal menstruation
32	Wu et al., 2004 [38]	Taiwan	Prospective non-randomized study (n = 40)	Total testosterone, LH, and LH/FSH ratio were shown to have significant decreases 3 months post-operation. The vascularization index and vascularization flow index of ovarian stroma were decreased post-LOD	LOD did not significantly affect leptin levels. However, ovarian stromal blood flow was shown to have changed post-LOD
33	Sorouri et al., 2014 [39]	Iran	Prospective randomized clinical trial study (n = 100)	There was a decrease in LH levels and testosterone levels in both ULOD and BLOD groups. FSH levels remained the same after LOD	ULOD was as effective as BLOD regarding both ovulation and pregnancy rates.
34	Mohammad, 2023 [40]	Iraq	Prospective cohort study (n = 75)	A statistically significant decrease in LH and AMH was found following LOD. LOD effectively improved fertility and menses regularity in a cohort of 75 infertile women	Laparoscopic drilling has a significant effect on decreasing AMH and LH in infertile women with PCOS. In PCOS, AMH can be used as a marker for LOD effectiveness
35	Giampaolino et al., 2017 [41]	Italy	Randomize controlled trial (n = 246)	In both LOD and THL-OD, there was a significant reduction in AMH levels by 17.5% and 17.3%, respectively, with no significant difference between the two types of OD. However, patients who underwent LOD had significantly more pain via the VAS score compared to those who underwent THL-OD	THL-OD may be a viable alternative to LOD with a similar reduction in AMH and significantly less pain
36	Sunj, et al., 2014 [42]	Croatia	Longitudinal cohort study (n = 96)	ULOD and BLOD demonstrated a decrease in AMH after surgery. However, this decrease was more profound in the BLOD group at 1 month follow-up. At the 6 months follow-up, a statistically significant increase in AFC and volume was noted in the ULOD group	There were no significant or long-term effects on ovarian reserve relative to dose-adjusted unilateral diathermy
37	Abuelghar et al., 2014 [43]	Eygpt	Prospective cohort study (n = 251)	Females with higher preoperative concentrations of LH and FSH were associated with higher rates of spontaneous ovulation, whereas ovarian volume was not	LH and FSH may be reliable predictors of LOD success
38	Fukuhara et al., 2014 [44]	Japan	Retrospective cohort study (n = 59)	The preoperative concentration of FSH was significantly higher in the participants who maintained spontaneous ovulation without further treatment	The preoperative concentration of SH was a significant predictor for the effectiveness of LOD

Risk of Bias in Included Studies

Using the GRADE scale as mentioned previously, and considering the procedures used throughout each study, and the populations, it was assessed whether replicability was seen across similar studies. The addition of case series also influenced this paper's overall risk of bias as their sample size was smaller and design more anecdotal. Overall, it was concluded that there was a medium risk of bias in the studies included in this review.

Synthesis of Results

Demographics: Across all studies, the average age of participants was 29.95 years, with an average BMI of 27.41 kg/m^2^; one study clarified that all patients in their study fell into phenotype A of PCOS [7-39]. In PCOS, enhanced male secondary sex characteristics such as hirsutism and acne were also seen, which were present in 43.2% and 49.1% of patients, respectively [8-10, 13, 34, 37]. Another problem associated with PCOS is the inability to conceive; 65.2% of patients with PCOS report primary infertility, and 34.8% reported secondary infertility [9, 15, 18, 25, 28, 40]. The average amount of punctures done during the procedure was 12.58 in each ovary across all studies (range: 3-120) [7-10, 13-17, 19,20, 22, 23, 27-30, 36, 41, 42].

Menstrual cycle pattern: Menstrual irregularities are common in patients with PCOS, and on average, 12.5% of patients had a regular menstrual pattern, 76.2% experienced oligomenorrhea, and 21% experienced amenorrhea [7-10, 13, 18, 19, 28, 32-34, 39]. In the first three months, Kandil et al. observed a 75.4% increase in average ovulation, which increased to 77.1%, in line with several other studies [10, 23, 35]; 79.5% of patients had cessation of oligomenorrhea, with an average of 73.9% experiencing spontaneous ovulation [8, 18, 21, 25, 29, 39]. Between unilateral and bilateral LOD methods, bilateral LOD had a higher number of participants undergoing spontaneous ovulation by 4.7% [39]. At three and six months following LOD, 16.22% and 54.06% of participants returned to a regular menstrual cycle, with Hafizi et al. recording up to 76.7% of their participants [18, 25, 33].

Kucuk and Kilic-Okman observed that 32% of their participants who previously experienced amenorrhea developed irregular menses following LOD [25]. Masroor et al. observed that 47% of participants who previously had oligomenorrhea continued to experience it following LOD [21]. Only one study classified LOD as unsuccessful due to the absence of spontaneous ovulation, and this was observed in 20% of their participants [29]. In patients who underwent unilateral LOD, 33.3% had resumption of menstrual periods through induction compared to 28.9% of patients who underwent bilateral LOD [39]. Al-Ojaimi's was the only other study to report trials of induction through human menopausal gonadotrophin, and they observed that 1.7% of participants remained anovulant [8]. Hamed et al. reported a total ovulation rate was 50.8% [19]. Variables such as inhibin B level, BMI, and age pre-LOD had no significant relation to previous and current menstruation cycles [9, 21].

AFC and ovarian reserve: In patients with PCOS, the average antral follicle count (AFC), which gives physicians an idea as to how many follicles are viable, of both ovaries was 18.97 with a median of 31 [12, 14, 18, 23, 37]. Api et al. reported that ultrasonographic evidence showed >10 follicles in each ovary at 6-8 mm in the peripheries [10]. Following bilateral LOD, there was a significant decrease in AFC, with an average of 25.95% across most studies [7, 8, 12, 18, 22-24, 31, 33, 34]. However, Sunj et al. observed no significant change in AFC one month post-op, and they only showed a slight initial decrease [42]. The right and left ovarian AFC decreased significantly by 41.85% and 43.01%, respectively, following LOD, with a slight association with amenorrhea and no association or correlation with hyperandrogenism, age, or BMI [7]. Compared to bilateral LOD, there was no significant difference in AFC levels following unilateral LOD, and it remained higher in unilateral LOD compared to bilateral LOD [24, 31]. Sunj et al., however, found a 650% increase in AFC at six months following unilateral LOD compared to the levels at one month [42].

Before LOD, studies reported an average ovarian volume of 11.33 cm^3^, with one study observing that the right ovarian volume averaged 12.2 cm^3^ and the left ovarian volume at 11.9 cm^3^ [9, 12, 14, 31, 37]. The ovarian volume also significantly decreased following bilateral LOD, by an average of 27.14% [12, 24, 38]. Compared to conservative treatment, the ovarian volume was 72% smaller following bilateral LOD [12]. Sunj et al. noted that these initial changes occurred during the first month and did not continue into the sixth month, which was not noted in other studies [42]. The right and the left ovarian volume decreased significantly by an average of 30.1% and 32.3%, respectively, and showed no significant correlation or association with hyperandrogenism or amenorrhea [7, 22]. Sunj et al. noted an increase in ovarian volume by almost 1000% between one and six months following unilateral LOD [42]. Regarding the overall health of the ovary, it was found that the flow index and vascularization index improved significantly, possibly contributing to spontaneous ovulation through the flow of hormones [22]. Overall, LOD can affect the ovarian reserve transiently; however, the reserve may decrease following bilateral LOD [23, 24].

Anti-Mullerian hormone: While it is clear that there is a decrease in the ovarian reserve and volume following LOD, there is some discrepancy in the alterations in AMH concentrations. The average amount of AMH present in patients with PCOS was 8.57 ng/mL, which is significantly higher than in healthy controls in most studies [7, 11-14, 16-18, 23, 27, 28, 30, 31, 33, 34, 37, 40, 41]. Hendriks et al. observed a significant and immediate decrease in AMH concentrations following LOD, whereas the majority of studies found it continued for six months with an average decrease of 36.92% [7, 11, 12, 14, 17, 20-23, 27, 29-31, 33, 34, 40-42]. Ogawa et al. observed that when compared to preoperative AMH concentrations, there was a 0.55% change in overall AMH concentrations per puncture in all patients. It is also noted that the percentage of AMH change is significantly increased in patients with a preoperative AMH level of >10 ng/mL, which correlates to an AMH concentration change of 66% per puncture [28]. There was also no significant difference between AMH concentration decrease and unilateral versus bilateral LOD [30, 31, 42]. However, four studies reported no significant change in AMH concentrations following LOD [13, 16, 18, 37].

There was a split between age and AMH concentration decrease, with one study reporting an association and two observing none [7, 28, 29]. Most studies also observed no association between BMI and AMH concentrations [7, 21, 23, 29]. However, one study extensively stratified age groups and found otherwise [28]. Participants with BMIs <25 kg/m^2^ had a significantly higher percentage decrease in AMH concentration compared to those with a BMI >25 kg/m^2^ [28]. Participants with a BMI <18.5 kg/m^2^ had the highest percentage decrease in AMH concentration, which correlated to a 1% change per puncture in this group, significantly higher than the other two BMI categories [28]. Abulkassem et al. reported a significant correlation between AMH concentrations and hyperandrogenism or amenorrhea [7]. Concerning menstrual cycle regulation, participants who were non-responders (no ovulation) had significantly higher AMH (>8.3 ng/mL) pre-LOD, which correlated to a 33.3% lower ovulation rate compared to the 100% ovulation rate in participants who had an AMH concentration <8.3 ng/mL [29]. Lastly, there was a significantly positive correlation between AFC count and AMH concentrations, tying back to the concept that AMH is naturally a good predictor of ovarian reserve [16]. However, the odds of AMH as a determinant of ovulation response significantly decreased one month following LOD [36].

Inhibin B: In PCOS patients, inhibin should theoretically be elevated. However, most concentrations charted reported an inhibin level of 54 pg/mL, which falls within the reference range [37]. Amer et al. reported that inhibin B has a significant inverse correlation with BMI compared to its association with PCOS [9]. Immediately following LOD, Hendriks et al. observed a significant decrease in inhibin B concentrations, which continued for five days following surgery [20]. This roughly correlates with two studies that found a significant decrease in inhibin B concentrations six months following LOD, with an average of 13.45%. However, three other studies observed no statistically significant difference in inhibin B levels following LOD [24].

Follicle-stimulating hormone: Compared to LH levels, FSH levels decrease in PCOS levels, with studies reporting a significant decrease in participants' FSH levels compared to healthy controls at 5.65 IU/L [7-10, 12-15, 17, 22, 24-26, 30-35, 38-40]. A few studies noted a significant increase in FSH concentrations in participants, with an average of 32.51% observed [7, 8, 10, 12, 35, 37]. Hendriks et al. observed an immediate and significant decrease in FSH following surgery; however, this did not persist and reached insignificant levels at follow-up, which correlated with the majority of studies [9, 14, 15, 17, 20, 29, 30, 33, 34, 38]. While Roy et al. also observed no significant changes after unilateral LOD, Rezaei et al. observed a significant increase in FSH levels [30, 32]. There was no significant difference between responders and non-responders in FSH concentration changes [8, 29]. Age, BMI, amenorrhea, and hyperandrogenism were not correlated or associated with FSH concentrations following LOD [7]. Two studies found that preoperative concentration of FSH was a significant predictor of the success of LOD [43, 44].

Luteinizing hormone: On average, the LH level was 11.54 IU/L, with Al-Ojaimi reporting that 62.9% of females in their study had elevated LH [8-10, 12-17, 19, 22, 23, 25, 26, 31-35, 38-40]. Due to the reduction in the ovarian reserve and AMH, LH should theoretically be decreased following LOD, as was reported in the majority of studies: there was an average reduction of LH in these studies at 36.35% with no significant difference between unilateral and bilateral LOD [8-10, 15, 19, 25, 26, 29, 32-35, 38-41]. However, several studies found no significant difference in LH values following LOD, but Hendrik et al. did notice an initial surge in LH five hours after LOD [12, 14, 16, 17, 20]. Differences between responders were split, with one study finding no significant difference in LH concentrations while two studies observed a significant reduction of LH in responders only [17, 29, 35]. Wu et al. evaluated the correlation between LH and BMI and found that the correlation between LH and leptin increased significantly by 120% following LOD. This correlates with the obese group only having a reduction of 33.46% in LH compared to 60.08% in the lean group following LOD [38]. No other studies observed correlations between BMI and LH, but further analysis should be done to improve LOD. However, it is crucial to note that the odds of LH as a determinant for ovulation response were increased following LOD and may prove to be a reliable predictor of success [34, 36, 43].

Luteinizing hormone/follicle-stimulating hormone ratio: In PCOS, with the increase in LH and decrease in FH, there is an increase in the LH/FSH ratio, which was documented to be at an average of 2.3 [8-11, 13-15, 22, 25-27, 33-35, 38]. Al-Ojaimi observed that the LH/FSH ratio was elevated in 45.9% of female participants [8]. Of note, 100% of studies that evaluated the LH/FSH ratio observed a significant decrease following LOD at an average of 38.89% [8-11, 15, 25-27, 29, 33-35, 38, 41]. Paramu observed no significant difference in the ratios between responders and non-responders [29]. As with LH, there was also a correlation between LH/FSH ratio and leptin, which increased by 58.29% following LOD. This correlated with the LH/FSH ratio decreasing by 31.82% in the obese group compared to 1.09% in the lean group following LOD [38]. While the change in concentration of FSH following LOD varied between studies, it was primarily evident that LH significantly increased.

Testosterone: Testosterone is typically increased in patients with PCOS and our review found it to be higher at an average of 3.24 nmol/L, with one study reporting a level as high as 11.68 nmol/L [8-10, 13-16, 18, 19, 22, 25, 32-35, 38, 39]. Testosterone levels were significantly more elevated in patients with PCOS compared to controls, with elevated levels reported in at least 29.8% of females [8, 37]. Due to the usually prevalent decrease in LH, testosterone should theoretically be reduced following LOD. Hendriks et al. observed an immediate and significant decrease in testosterone following LOD that continued into day five [20]. At six months following LOD, the majority of studies observed a significant decrease in testosterone concentration with an average of 31.05% [8-10, 15, 18, 19, 25, 29, 33-35, 38, 39].

This occurrence was also replicated with unilateral LOD, which had an average decrease of 36.31% [32, 39]. Three studies, however, observed no significant difference in testosterone concentrations following LOD [14, 16, 37]. No significant difference was observed when LOD was performed through dose-adjusted measures [18]. Paramu noted that testosterone concentrations were significantly higher preoperatively in non-responders than responders and positively correlated with AMH concentrations [29]. Wu et al. noted a correlation between total testosterone and leptin, which increased significantly by 140% following LOD. This correlates to a total testosterone reduction of 15.84 in the obese group and 26.67% in the lean group following LOD [38]. Following LOD, there was a decrease in the odds of testosterone being an indicator of ovulation response, which correlates with the significant percentage of participants who developed spontaneous ovulation [36].

Discussion

Principal Findings

One of the main clinical criteria for PCOS is the clinical diagnosis of anovulation, which inherently affects the fertility rate. PCOS also includes polycystic ovaries, which are not only created by a hyperandrogenic state but promote this state through LH. AMH also plays a role in the ovulation and implantation of the fertilized egg, and it may also determine the success of LOD. LOD is often used as a last resort, ultimately to enable females to conceive; however, to do this, there needs to be some regulation in their menstrual patterns. LOD mechanically manipulates the ovaries and eggs within, which correlates to the reduced ovarian reserves concerning AFC and volume. This reduction leads to a decrease in the production of AMH and should decrease inhibin as the ovarian follicles produce these hormones; however, AMH was the only parameter to show a reduction.

It is unclear what the non-significant changes in inhibin B equate to, but it is in line with the lack of differences noted regarding FSH concentrations, which should have increased. LH, which contributes significantly to ovulation and the menstrual cycle's luteal phase, decreased significantly. This is consistent with spontaneous ovulations, as polycystic ovaries are penetrated by the drill, inhibiting its overproduction of LH and disrupting non-cystic ovaries. Significantly high levels of LH and significantly low levels of FSH may be predictors of LOD success, as this alignment of hormones allows for ovulation. A decrease in LH correlates with a decrease in testosterone as LH functions to increase androgen synthesis and concentrations. All of these contribute to the high amounts of spontaneous ovulation observed in studies that tracked this outcome.

Comparison With Existing Literature

While LOD has been performed for over two decades, its pathomechanism is still not fully understood. It is known that destroying the ovarian cortex and stroma tissue reduces androgenic follicles [45]. This review highlights that the androgenic follicles are the antral follicles, the reserves of the ovary, which is why certain hormone alterations may be observed. AMH has been found on numerous occasions, including in this review, to be significantly reduced following LOD, but it is unclear if this is due to actual damage [46]. One study observed that LH and FSH significantly increased shortly after LOD for the initial two days, followed by a persistent decline in LH concentrations [45, 46]. FSH remains a matter of controversy in the literature in general and studies included in this review. The same goes with inhibin, as few reports of its concentration changes have been found in the literature. One aspect not observed in these studies is the changes in estrogen levels due to the destruction of the androgenic follicles. Strowitizki et al. reported that there should also be a decrease in the peripheral conversion of androgens to estrogens; however, no study focused on this possible effect [45]. The changes observed in these hormones align with the spontaneous or improved ovulation cycle in females with PCOS [45-47].

Strength and Limitations

A major strength of this study is that we were able to compare the hormones across studies to track possible correlations based on the theoretics of this treatment modality. While we can conclude a uniform result, we can interpret that the treatment seems to follow expectations. Another strength pertains to the design of this review; we conducted a rather broad search to elucidate any hormone variation following LOD that may contribute to the return of spontaneous ovulations. This allowed for a concise algorithm to include all articles and narrow down the amount with the participation of four coauthors to reduce bias and determine the true impact of the results in the articles included.

This study has a few limitations. Primarily, the hormones were so spread out that there was no study continuity, and loose interpretations were based on inter-study connections, hindering the interpretation's impact. It is important to note that some studies did not describe the assay used to assess AMH, and it is known that the range of AMH varies significantly between the different assays. With that said, all studies that found a significant difference were used together, and the average obtained was based on those with the same assays. Hence, despite the varying numbers, the results are the same. The lack of studies on FSH and inhibin B can also considered be a limitation as FSH is the primary hormone in the follicular stage of the menstrual cycle, affecting menstrual regularities. It would be better to understand the relationship between inhibin B and FSH to correlate known pathophysiology to the alterations done through mechanical manipulation such as LOD. Finally, another limitation of this study is that it mostly focused on the Eastern region and included only a few studies involving Western regions. With the growing rate of PCOS in Western cultures, it is critical to analyze how individuals in these cultures are affected by this condition.

## Conclusions

PCOS is an intricate endocrine/reproductive condition, and its incidence is on the rise globally. It affects multiple aspects of a patient’s life, most prominently the ability to have a normal menstrual pattern and the ability to conceive. This review evaluated the former, including the ovarian reserve, AMH, inhibin B, LH, FSH, LH/FSH ratio, and testosterone, as they are the commonly altered hormones in PCOS. LOD was robustly successful in inducing spontaneous ovulation, and in most cases where ovulation did not return spontaneously or returned in an irregular state, ovulation could be caused by exogenous hormones, making LOD an appropriate last-resort treatment modality. Future research should focus a little more on the FSH and inhibin B alterations and interplay and how they may also contribute to ovulation resumption and solidify what these alterations are. Lastly, the inability to conceive is a major problem for most couples, and LOD has been shown in a few cases to lead to an increase in pregnancy rates. Further research should not only track the pregnancy rate associated with the use of LOD but also the effect on pregnancy, such as multiple gestations, as well as obstetric, maternal, and neonatal complications.
